# Influencing factors of low vision 2 years after vitrectomy for proliferative diabetic retinopathy: an observational study

**DOI:** 10.1186/s12886-023-03071-4

**Published:** 2023-07-10

**Authors:** Shengxia Wang, Yongjun Liu, Yunhong Du, Huijing Bao, Junli Zhu, Xin Liu

**Affiliations:** 1 Department of Ophthalmology, , The Affiliated Taian City Central Hospital of Qingdao University, No.29 Longtan Road, Taian, 271000 Shandong People’s Republic of China; 2 School of Clinical and Basic Medicine, Shandong First Medical University (Shandong Academy of Medical Sciences), Taian, 271000 Shandong People’s Republic of China

**Keywords:** Pars plana vitrectomy, Proliferative diabetic retinopathy, Low vision, Surgical complication, Anti-VEGF

## Abstract

**Background:**

Proliferative diabetic retinopathy (PDR) can seriously affect the vision and quality of life of patients. The present study aimed to evaluate the clinical effect of vitrectomy for PDR by observing visual recovery and postoperative complications and to explore the factors influencing low vision.

**Methods:**

This was a case series observational study. Consecutive eyes of patients with PDR who underwent 23G vitrectomy in our hospital within one year (2019.11-2020.11) were collected and followed up for more than 2 years. Patients’ visual acuity, surgical complications and management were collected before the operation and during the follow-up. Decimal visual acuity was recorded and converted to the logarithm of the minimal angle of resolution (logMAR) for statistical analysis. Excel was used to establish a database, and SPSS 22.0 statistical software was used for data analysis.

**Results:**

A total of 127 patients and 174 eyes were included in the study. The mean age was 57.8 years. The best corrected visual acuity (BCVA) was < 0.3 in 89.7% of eyes before surgery and ≥ 0.3 in 48.3% of eyes after surgery. Among 174 eyes, visual acuity improved in 83.3%. There was no change in 8.6% of eyes, while 8.1% of eyes had decreased visual acuity after surgery. The average logMAR visual acuity was 1.5 ± 0.7 before surgery and 0.7 ± 0.6 after surgery, indicating significant improvement (*p* < 0.05). Logistic regression analysis showed that intraoperative silicone oil filling and postoperative complication were significant risk factors for postoperative low vision, while preoperative pseudophakic lens and postoperative intra vitreal injection of anti-VEGF were protective factors for vision recovery (*p* < 0.05). The incidence of postoperative complications was 15.5%, top three of which were vitreous haemorrhage, neovascular glaucoma and traction retinal detachment.

**Conclusion:**

Vitrectomy is safe and effective in the treatment of PDR with few complication. Postoperative intra vitreal injection of anti-VEGF is a protective factor for vision recovery.

**Trial registration:**

The trial registration number is ChiCRT2100051628, and the date of registration was September 28, 2021.

## Background

Diabetic retinopathy (DR) is the leading disease-based cause of blindness among the working-age population [1]. Proliferative diabetic retinopathy (PDR) is the most severe ocular disease in patients with type 1 and type 2 diabetes, and it can cause serious damage to visual function. Using the diagnostic criteria of the American Diabetes Association in 2018, Li et al. conducted a survey on 75 880 Chinese adults aged ≥ 18 years. The survey found that the total prevalence of diabetes mellitus in 2017 was 12.8%, equivalent to roughly a total of 129.8 million patients of diabetes mellitus in China [2]. Yang et al. performed a systematic review and meta-analysis of 48 995 patients with type 2 diabetes mellitus in Asia, revealing 25% prevalence of DR, with PDR accounting for 15% [3].

Vitreous haemorrhage (VH), neovascularization, and retinal detachment caused by PDR require appropriate pars plana vitrectomy (PPV). PPV surgery is a crucial treatment for preserving the eyesight of PDR patients, as it can clear both VH and proliferative membranes. In addition, intraocular panretinal photocoagulation (PRP) can be performed alongside PPV surgery to improve retinal ischaemia.

However, the visual acuity, risk factors and incidence of surgical complications reported in recent clinical studies using different design methods vary in different regions. Additionally, the included subjects often span many years in operation time and surgical methods rang from 20G to 25G PPV [4, 5]. Thus, in this study, we observed consecutive 23G standard three-channel closed PPV procedures performed by the same surgeon in our hospital within one year, focusing on postoperative vision recovery and surgical complications and analysing the influencing factors of postoperative low vision.

## Methods

This was a case series observational study. The study was performed in accordance with the Declaration of Helsinki and was approved by the ethics committee of Taian City Central Hospital [approval number: 2021-06-82]. The trial registration number is ChiCRT2100051628, and the date of registration was September 28, 2021. Patients with PDR who underwent 23G PPV in Taian City Central Hospital from December 2019 to November 2020 were included in the study. The specific inclusion criteria were as follows: PDR treated with 23G minimally invasive PPV due to refractory vitreous haemorrhage (VH), traction retinal detachment (TRD) involving macula, or TRD with retinal tear. The exclusion criteria were as follows: previous history of vitrectomy; blindness due to trauma or other eye diseases; and patients who were lost to follow-up for more than half a year. The collected data included demographics, visual acuity at baseline and follow-up, complications, and treatment required during follow-up. Low vision and blindness were defined as visual acuity between 0.3 and 0.05 and visual acuity worse than 0.05, respectively (World Health Organization [WHO] criteria) [6]. An intra vitreal injection of anti-VEGF (Ranibizumab[10 mg/ml]/Aflibercept[40 mg/ml], 0.05 ml) was administered 3–7 days before surgery. The same experienced surgeon performed all operations. All procedures were performed under retrobulbar nerve block anaesthesia, except for one patient with Alzheimer’s disease who was unable to cooperate and required general anesthesia for the surgery. A Lumera S88 from Zeiss was used as the surgical microscope, and a Constellation from Alcon USA was used for the vitrectomy. A 23G surgical kit and RESIGHT LH200 wide-angle lens were used. Intraoperative retinal photocoagulation was performed approximately 1200 ~ 1600 points. Intraoperative vitreous cavity fillers (balanced salt solution/sterile air/silicone oil) were selected according to the severity of PDR in the affected eye. The patients returned to the ophthalmic clinic for reexamination 1 week, 2 weeks, and 1 month after surgery. Fluorescein fundus angiography (FFA) was performed 3 months after surgery if necessary. If retinal capillary nonperfusion zone (NP) was found, supplementary retinal photocoagulation therapy would be given in time to avoid further VH or NVG. During the follow-up period, necessary treatments were given to both eyes according to the patient’s condition, including laser supplementation for local NP or mild VH, intra vitreal injection of anti-VEGF (Ranibizumab[10 mg/ml]/Aflibercept[40 mg/ml], 0.05 ml) for macular oedema involving the macular centre and recurrent VH, phacoemulsification and intraocular lens implantation for cataracts with visual impairment, and PPV for recurrent VH and TRD. All patients completed at least two years of follow-up, with a mean follow-up time of 27.9 months. Decimal visual acuity was recorded and converted to the logarithm of the minimal angle of resolution (logMAR) for statistical analysis. Excel was used to establish a database, and SPSS 22.0 statistical software was used for data analysis. A comparison of continuous variables was performed utilizing Student’s t test, while categorical data were analysed using the chi-squared test. The risk factors for postoperative visual acuity and complications were analysed by logistic regression. A *p* value of < 0.05 was used to determine statistical significance.

## Results

### Demographic data and visual acuity

A total of 191 eyes of 140 patients underwent surgery during this period, and 127 patients met the inclusion criteria and were included in the study, including 47 patients undergoing binocular surgery and 80 patients undergoing monocular surgery, for a total of 174 operations.

Among the 127 patients included in the study, the mean age was 57.4 ± 9.7 years (range 24–78 years). The mean self-reported diabetes duration was 9.5 ± 6.1 years (range 1–38 years). The mean HBA1c was 7.5 ± 1.7 (range 4.9–13.7). Further details on characteristics of the classified data are shown in Table 1.

**Table 1:** Classification data characteristics of operation cases

Index	classification	n	%
Gender	male	64	50.4
	female	63	49.6
Type of diabetes	type1	3	2.4
	type2	124	97.6
Combined heart disease(%)		37	29.1
Combined nephropathy(%)		9	7.1
Combined hypertension(%)		62	48.9
Indications for PPV (%)	VH	134	77.0
	TRD	36	20.7
	dense macular hemorrhage	3	1.7
	VH after PRP	1	0.6
Contralateral eye DR Grading	Mild NPDR	5	6.3
	Moderate NPDR	24	30.0
	Severe NPDR	35	43.7
	PDR	16	20.0
Preoperative lens state	Crystalline lens	153	87.9
	Pseudophakic	21	12.1
Postoperative lens state	Crystalline lens	86	49.4
	Pseudophakic	37	21.3
	Aphakia	51	29.3
PRP before operation		37	21.3
Anti VEGF after operation		40	23.0
Intraoperative filling	balance salt solution	95	54.6
	sterile air	56	32.2
	silicone oil	23	13.2
Postoperative complications(%)		27	15.5

Indications for surgery: Among the 174 surgical cases, 77.0% were due to refractory vitreous haemorrhage, 20.7% were due to traction retinal detachment caused by proliferation, and the remaining surgeries were due to dense macular haemorrhage (1.7%) and repeated vitreous haemorrhage after retinal laser photocoagulation (0.6%). Almost two-thirds (63.7%) of the contralateral eyes had severe nonproliferative diabetic retinopathy (SNPDR) and PDR. Intraocular PRP therapy was performed in 78.7% of eyes. More than half of the affected eyes (54.6%) were filled with balanced salt solution, 32.2% with sterile air and 13.2% with silicone oil. Silicone oil was removed 3–6 months after surgery. A total of 12.1% of patients had pseudophakic lens before the operation. Cataract extraction and intraocular lens(IOL)implantation were performed in 16 cases, while fifty-one needed secondary IOL implantation because accurate IOL power could not be measured at that time.

During follow-up, 28 patients received monthly intra vitreal injection of anti-VEGF for at least three consecutive months, including 40 surgical eyes. Retinal supplementary photocoagulation was performed in 16 surgical eyes and PRP in 56 nonsurgical eyes.

**Fig. 1:**
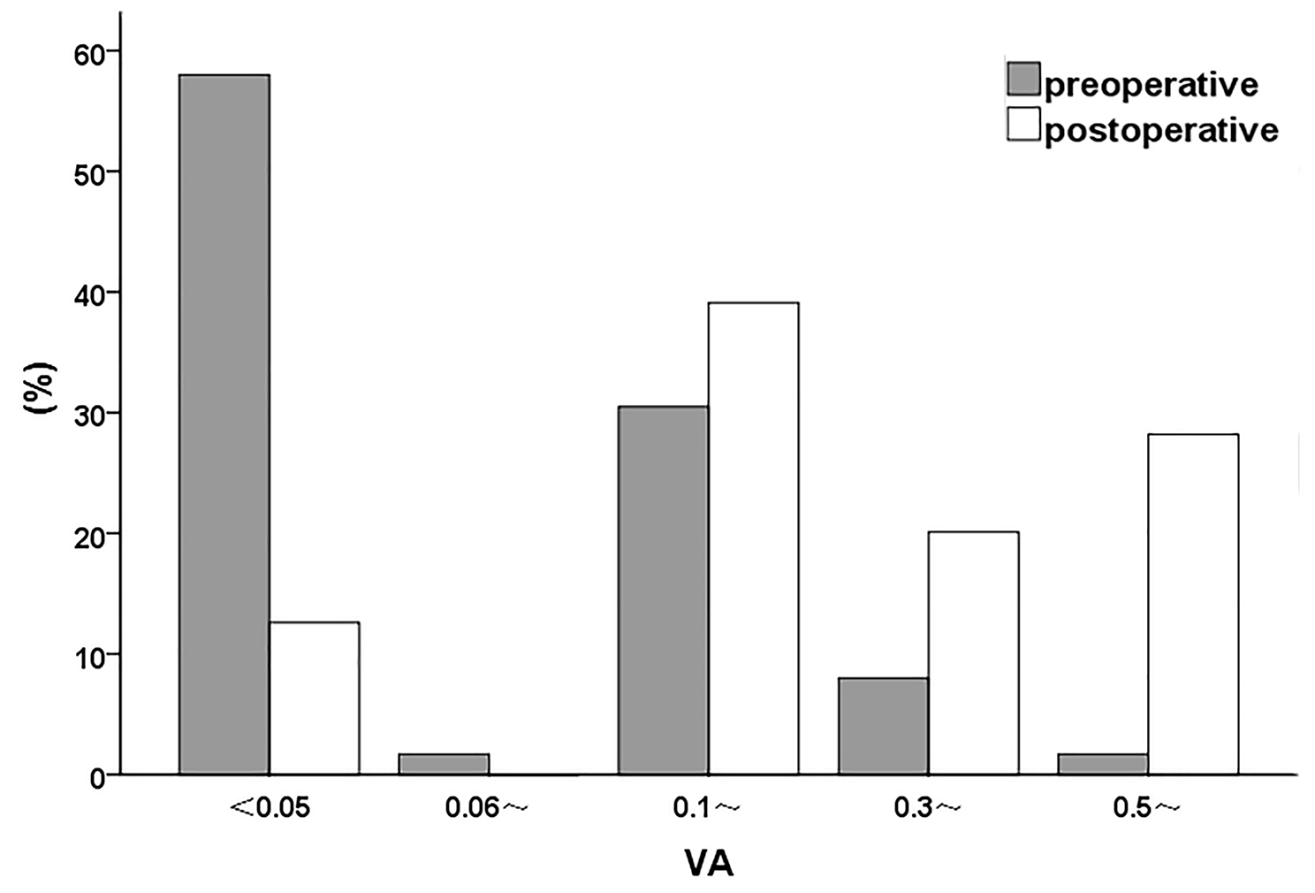
Percentage of segmented visual acuity before and after surgery (%)

Preoperative BCVA ranged from light perception (LP) to 0.6, while BCVA < 0.05 was observed in 52.3% of eyes, and BCVA < 0.3 was observed in 89.7% of eyes. Postoperative BCVA ranged from hand movement (HM) to 1.0, while BCVA ≥ 0.05 was observed in 90.2% eyes, and BCVA ≥ 0.3 was observed in 48.3% eyes. Among 174 eyes, visual acuity improved in 83.3% of eyes. There was no change in 8.6% of eyes, while 8.1% of eyes had decreased visual acuity after surgery. The average logMAR visual acuity was 1.5 ± 0.7 before surgery and 0.7 ± 0.6 after surgery. The postoperative visual acuity was significantly improved (*p* < 0.05). The segmented comparison of preoperative and postoperative visual acuity is shown in Fig. 1.

### Analysis of risk factors for postoperative low vision

Postoperative visual acuity was used as the dependent variable and was divided into two categories according to the BCVA (≥ 0.3 or < 0.3). Logistic regression analysis was performed on age, diabetes course, HbA1c, contralateral DR grade, postoperative complications, etc. The value of dependent variable BCVA ≥ 0.3 was set as 0, while BCVA < 0.3 was set as 1. The OR value indicates the risk of a variable; a value greater than 1 indicates that the variable is a risk factor, whereas an OR less than 1 indicates that the variable is a protective factor.

Among the variables included in the regression equation, intraoperative silicone oil filling and postoperative complications were the most significant risk factors for postoperative low vision (*p* < 0.05), while a high HBA1c level (*p* = 0.051) and high contralateral eye DR grade (*p* = 0.063) were potential risk factors. Preoperative pseudophakia and postoperative intra vitreal injection of Anti VEGF were protective factors for postoperative visual acuity (*p* < 0.05).

The logistic regression analysis results of the risk factors for postoperative low vision are shown in Table 2.

**Table 2:** The logistic regression analysis of risk factors for postoperative low vision

	P值	OR	95%CI
HbA1c(%)	0.051	2.561	0.996–6.588
contralateral DR grade	0.063	1.629	0.974–2.724
preoperative lens status	0.022	0.17	0.037–0.779
postoperative anti VEGF	0.068	0.35	0.113–1.08
intraoperative filling	0.002	2.792	1.466–5.318
complications	0.031	5.457	1.166–25.537

OR: Odds ratio; CI: Confidence interval; HbA1c(%)Glycosylated hemoglobin

Twenty-seven eyes had postoperative complications among the 174 operations. There were fourteen eyes with postoperative VH. Among them, eight patients required reoperation with PPV, and four of them underwent more than two reoperations. Six eyes had haemorrhage absorption after drug treatment, including intra vitreal injection of anti-VEGF and oral administration of He Xue Ming Mu Pian and Luan Lin Zhi Luo He Dian. He Xue Ming Mu Pian is a kind of traditional Chinese medicine which can nourish the liver and protect the eye. It’s used as an auxiliary treatment for VH. Neovascular glaucoma (NVG) developed in five eyes and was treated with anti-VEGF injection followed by filtration surgery or supplementary laser therapy. Ocular massage and carteolol and brinzolamide eye drops were applied to lower intraocular pressure during the follow-up. At the end of follow-up, four cases had stable intraocular pressure, while one case had poor control of intraocular pressure, resulting in no light perception (NLP). TRD was found in three eyes. All three eyes were operated on smoothly and filled with sterile air. However, TRD was found 1 month after surgery. The re-proliferative epiretinal membrane was removed by a second PPV. During the re-operation, the traction of the ERM was released and retinal photocoagulation was supplemented. Finally, silicone oil was filled to aid retinal reattachment. Unfortunately, the retina was not reattached after removal of the silicone oil in two eyes. Therefore, we performed another operation. Silicone oil filling combined with external scleral ligation was performed to assist the retina reattachment. The time of oil extraction was extended to half a year after the operation, and retinal reattachment was good at the end of follow-up. There were two cases of macular preretinal membrane and one case of macular hole, in which the anatomical structure recovered well after the reoperation. There was one case of secondary glaucoma after silicone oil filling. The anterior chamber was filled with silicone oil resulting in elevated intraocular pressure. A small amount of silicone oil was released through anterior chamber puncture under surface anaesthesia. The patient recovered after treatment in the strict face-down positioning and carteolol and brinzolamide eye drops. Postoperative optic atrophy occurred in one case. Curiously, this patient did not develop Ocular hypertension during follow-up despite intraoperative silicone oil filling. BCVA was finger counting (FC) after removal of silicone oil. Optical coherence tomography (OCT) showed significant thinning of the optic nerve fibre layer, which may be attributed to the silicone oil toxicity on retina and optic nerve [9, 10].

Logistic regression analysis showed that among the variables included in the regression equation, the grade of diabetes duration was a significant risk factor for postoperative complications (*P* < 0.05). Duration of diabetes mellitus was recorded as the number of patient-reported years of disease. According to the duration of disease, it was divided into three grades: 1–5 years, 5–10 years, and more than 10 years. The higher the grade of the diabetes duration was, the greater the possibility for postoperative complications. Meanwhile, postoperative intra vitreal injection of anti-VEGF was a significant protective factor (*p* < 0.05). The results of logistic regression analysis of risk factors for postoperative complications are shown in Table 3.

**Table 3:** The logistic regression analysis of risk factors for postoperative complications

	P value	OR	95%CI
duration of diabetes mellitus	0.057	1.29	0.992–1.678
grade of diabetes duration	0.022	0.065	0.006–0.678
postoperative anti-VEGF	0.028	4.077	1.168–14.226

## Discussion

Among the 174 eyes analyzed in this study, 83.3% had improved visual acuity after surgery, of whom 90.2% had a BCVA ≥ 0.05, and 48.3% had a BCVA ≥ 0.3. Our findings demonstrate significant improvement in postoperative visual acuity, which is consistent with the results of other studies [4, 5].

Logistic regression analysis showed that intraoperative silicone oil filling and postoperative complications were significant risk factors for postoperative low vision. In addition, high HBA1c levels (*p* = 0.051) and contralocular DR grade (*p* = 0.063) were identified as potential risk factors. All these factors correlated with the severity of PDR. High HBA1c levels indicate poor recent blood glucose control, while high contralateral DR grade represents poor retinal function in both eyes [4]. Intraoperative silicone oil filling is a frequently employed intervention for severe PDR. Furthermore, patients with poorly controlled blood glucose levels and severe PDR had an increased risk of postoperative complications.

Vitreous cavity filling with silicone oil can help reattach the retina, stabilize the retinal structure, limit retinal bleeding, prevent low intraocular pressure, etc. [7]. However, it can also cause various complications, including cataracts, corneal degeneration, silicone oil emulsification, secondary glaucoma and silicone oil retinotoxicity [8–10]. In our study, silicone oil was only used to treat severe cases of proliferative traction retinal detachment or retinal holes, accounting for 13.5% of all operations. These patients typically have poor retinal function and a negative visual prognosis. When the retina is flat, the laser spot responds well, and the hole is closed and stabilized, the silicone oil is removed within 3–6 months to reduce the incidence of related complications. Although we found an association between intraoperative silicone oil filling and postoperative poor visual acuity, this does not mean that intraoperative silicone oil use should be reduced. The decision of whether to use silicone oil filling should be made by the surgeon based on the status of the retina during surgery.

Postoperative low vision is often caused by persistent proliferative folds of the retina, macular ischaemia, macular cystoid oedema, or photoreceptor damage. Our study confirmed that postoperative complications were associated with postoperative low visual acuity. Similarly, Nishi K [5] reported improved postoperative visual acuity among individuals without complications two years after PDR surgery. In the present study, the incidence of postoperative complications was 15.5%, with VH and NVG being the two most common complications observed. Additionally, there were three cases of TRD, two cases of macular preretinal membrane, one case of macular hole, and one case of optic nerve atrophy. Although the number of these cases was small, the visual impairment was significant.

Postoperative VH is a common complication after PPV in patients with PDR, and it is also a crucial factor affecting their visual prognosis [11]. In this study, the incidence of postoperative VH was 8.0%, occurring between 1 week to 21 months after surgery. This incidence is significantly lower than the 9.2%~32.4% reported in previous studies [12, 13]. The reduction in postoperative VH incidence can be attributed to two primary reasons. Firstly, with the development of minimally invasive vitrectomy technology and equipment [14, 15], PPV surgery has become safer and more efficient, resulting in reduced intraoperative bleeding and more thorough clearing of the basal vitreous body. Moreover, PRP applications have also become more extensive, alleviating peripheral retinal ischemia and hypoxia. Secondly, preoperative intra vitreal injection of anti-VEGF has contributed to the decrease in postoperative VH incidence. A new meta-analysis [16] including only randomized controlled trials showed that preoperative intra vitreal injection of anti-VEGF can reduce the incidence of postoperative VH, which is consistent with previous retrospective clinical studies.

NVG is also a common complication after PPV treatment of PDR. In this study, the incidence of postoperative NVG was 2.9%, significantly lower than the 4.6% ~ 19.4% reported in previous studies [17–19]. This may be related to preoperative intra vitreal injection of anti-VEGF. Lu et al. [20] suggested that preoperative anti-VEGF could not only help reduce the difficulty of surgery and the amount of intraoperative and postoperative bleeding but also reduce the occurrence of postoperative NVG. A 12-month follow-up study of 156 PDR patients after PPV showed that the incidence of NVG was 1.7% in the intra vitreal injection of anti-VEGFgroup before surgery, which was significantly lower than the 12.4% in the group without anti-VEGF injection before surgery. Furthermore, FFA was performed three months after the operation if necessary, and retinal photocoagulation was promptly provided in the retinal capillary nonperfusion zone. Retinal photocoagulation can permanently destroy the ischaemic retina, improve the state of retinal ischaemia and hypoxia, and prevent the generation of retinal neovascularization [21].

The present study has also discovered that postoperative intra vitreal injection of anti-VEGF serve as a protective factor for postoperative complications and visual recovery. Postoperative intra vitreal injection of anti-VEGF can assist in reducing macular edema, which leads to an improvement in vision, and it can also mitigate the ischemic and hypoxic state of the retina, thus attenuating the creation of retinal neovascularization. As VEGF is a cytokine that plays a prominent role in neovascularization within the eye, elevated levels of VEGF may persist in the eyes of PDR patients even after PPV, which could contribute to an increased risk of PVH, NVG and other complications [18, 20]. Therefore, postoperative anti-VEGF treatment is essential. Multiple randomized controlled clinical studies [22–24] have demonstrated that anti-VEGF treatment can improve vision in PDR patients without baseline macular oedema at 1, 2, and 5 years without increased endophthalmitis or cardiovascular events. In our clinical treatment, we apply it to patients with postoperative macular edema and recurrent vitreous hemorrhage. Typically patients received monthly intra vitreal injection of anti-VEGF for at least three consecutive months. Thereafter, PRN treatment was given based on investigator assessment. However, it needs to be explored whether intra vitreal injection of anti-VEGF can be routinely utilized in patients after PDR through multicenter and large-sample clinical studies.

Furthermore, this study revealed that the status of IOL before PPV serves as a protective factor for vision recovery, primarily because that such patients have elevated requirements for visual function and can detect vision problems as early as possible. In addition, the removal of the basal vitreous body can be performed more conveniently and thoroughly during the PPV surgery to reduce the risk of postoperative complications.

This study has certain limitations. As a retrospective study, it did not utilize a randomized controlled design. Nevertheless, the study also exhibits several advantages, including a high number of surgeries, a long-term follow-up period, and a standardized surgical procedure performed by a single surgeon with identical surgical equipment over the course of one year. This minimized variations in surgical technique among different operators. Furthermore, the study provides significant insights into the characteristics of real-world cases specific to this region.

## Conclusions

In summary, PDR is a serious eye disease that can lead to blindness. This study found 23G PPV to be a safe and effective method in treating PDR, with considerable postoperative visual acuity improvements and limited surgical complication in most patients. Postoperative complication is the most significant risk factor for postoperative low vision while regular postoperative intra vitreal injection of anti-VEGF is an important protective factor for vision recovery. Appropriate application of anti-VEGF and proper management of postoperative complication are particularly important.

## Data Availability

All data generated or analysed during this study are included in this manuscript. The raw data of the current study is available from the corresponding author on reasonable request.

## List of abbreviations

BCVA: Best corrected visual acuity
DR: Diabetic retinopathy
FC: Finger counting
FFA: Fluorescein fundus angiography
HM: Hand movement
log MAR: Logarithm of the minimal angle of resolution
LP: Light perception
NP: Nonperfusion zone
NVG: Neovascular glaucoma
OCT: Optical coherence tomography
PDR: Proliferative diabetic retinopathy
PPV: Pars plana vitrectomy
PRP: Panretinal photocoagulation
SNPDR: Severe nonproliferative diabetic retinopathy
TRD: Traction retinal detachment
VEGF: Vascular endothelial growth factor
VH: Vitreous haemorrhage
WHO: World Health Organization
